# Odontogenic Head and Neck Region Infections Requiring Hospitalization: An 18-Month Retrospective Analysis

**DOI:** 10.1155/2021/7086763

**Published:** 2021-01-18

**Authors:** Ewa Zawiślak, Rafał Nowak

**Affiliations:** Department of Maxillofacial Surgery, Silesian Piast Medical University, Borowska 213, Wrocław 50-556, Poland

## Abstract

The aim of this study was to comprehensively review our experience with odontogenic infections in the head and neck region requiring treatment at a national referral center. We retrospectively reviewed 85 patients treated at the Chair and Clinic of Maxillofacial Surgery of the University Hospital in Wrocław between January 2018 and June 2019. We excluded patients with nonondontogenic infections or other than purulent clinical forms of dentivitis in the head and neck region. Several demographic, clinicopathological, and treatment variables were assessed. The majority of patients were men who were referred for inpatient treatment by a dentist or family doctor, presented to the Hospital Emergency Ward (SOR) by themselves, or transported to the SOR by paramedics SOR from their home or another hospital. All patients were treated in accordance with the current guidelines for head and neck region odontogenic infections. An incision was made and the abscess was drained. The odontogenic cause was removed followed by the collection of tissue for microbiological examination. The course of infection was monitored by means of laboratory parameters such as leukocyte counts and c-reactive protein levels. Odontogenic infections in the head and neck region are a persistent and common problem. Rapid, accurate diagnosis and treatment minimizes the risk of life-threatening complications, shortens the hospitalization period, and lowers treatment costs.

## 1. Introduction

Despite universal access to dental care and antibiotic therapy, odontogenic infections requiring hospitalization remain a serious clinical problem [1–3]. Bacterial virulence, decreased host immunity, and lack of appropriate treatment lead to the spread of localized odontogenic infections to the deep fascial spaces of the head and neck [4, 5]. Odontogenic infections in the head and neck region may cause life-threatening complications, including respiratory obstruction, diffuse inflammatory abscess processes, necrotizing fasciitis, purulent meningitis, cerebrospinal abscesses, mediastinitis, sepsis, and septic shock [6–9].

The infections spread via the smallest tissue resistance point. The natural anatomical barriers to their spread are the cortical bone, periosteum, and the head and neck fascia system. Moreover, muscle and ligament attachments and the starting point of the infection (jaw or mandible teeth) play a key role in its localization [10]. The course of the infection and its clinical form depends on the patient's immune status, type and virulence of the microorganisms, and the treatment administered [11–13]. The treatment of choice for odontogenic infections is the incision and drainage of the abscess and possible removal of the odontogenic infection source in the oral cavity, if present. The optimal sequence of surgical procedures remains open to discussion [14]. Generally, it is recommended to remove the infection source (usually the causative tooth) during incision and drainage, if the patient's condition allows it [6, 15, 16]. Early diagnosis and appropriate treatment are crucial for quick recovery, shortening the hospitalization period, and reducing the risk of severe systemic complications [5, 9, 17, 18]. Systemic treatment includes empirical antibiotic therapy according to the antibiogram. Material for microbiological examination is collected during the abscess drainage in order to identify the microbial species, their sensitivity to specific groups of antibiotics and chemotherapeutics (antibiogram), and to determine the minimum inhibitory concentration (MIC). Determining the MIC helps plan the effective drug dose [19–22]. Nonsteroidal anti-inflammatory drugs and paracetamol are commonly used for supplementary therapy [23, 24]. The monitoring of body temperature, arterial blood pressure, and laboratory markers of inflammation (leukocytosis), c-reactive protein (CRP) level, and possibly the level of procalcitonin is obligatory [24–26]. The clinical picture of deep fascial infections of the head and neck may not correlate with the worsening of the patient's general condition. The standard diagnostic imaging approach involves computed tomography with contrast [27–29], which allows for precise location and differentiation of inflammatory disease from other pathologies, such as cysts, tumors, and metastatic lymph nodes [30].

The aim of this study was to investigate the predisposing factors, etiologies, clinicopathological and microbiological features, and treatment of odontogenic infections of the head and neck. Moreover, we reviewed current morbidity outcomes according to gender, age, and season.

## 2. Material and Methods

This retrospective epidemiological analysis was based on a review of the medical records of patients treated at the Department of Maxillofacial Surgery, Wroclaw Medical University Hospital. The study involved 85 patients hospitalized for odontogenic infections in the head and neck between January 2018 and June 2019.

The characteristics analyzed included age, gender, location of the infection, size of the fascial spaces occupied, laboratory markers of inflammation (leukocyte counts and CRP levels), type of anesthesia used, causal tooth group, hospitalization duration, seasonality, and type of cultured microorganisms.

The diagnosis was made on the basis of the history of the patient, clinical examination, and X-ray or CT scans imaging. Therefore, the patients were given a broad-spectrum empiric antibiotic therapy.

All of them underwent surgical treatment including drainage of inflammation infiltration under local or general anesthesia.

Routine blood tests and bacteriological examinations of the inflammation drainage were carried out.

The reference ranges for standard values at our laboratory were 4 × 10^3^–10 × 10^3^/mm^3^ for the white blood cell count (WBC) and less than 0.5 mg/dL for the c-reactive protein (CRP).

The appropriate characteristics of the descriptive data are reported as medians, ranges, and percentages.

The general health status and the presence of underlying systemic diseases were not recorded.

The patients with head and neck cancer, nonodontogenic head and neck infection, and posttraumatic infections were excluded from the study.

Statistical analyses were conducted using STATISTICA v. 13 (StatSoft, Inc., Tulsa, OK). The normality of all quantitative parameters was checked using the Shapiro-Wilk test. For all measurable parameters, the mean values (*M*), standard deviations (SD), medians (Me), lower (*Q*_1_), and upper (*Q*_3_) quartile were calculated along with the smallest (Min) and largest (Max) values. The intergroup differences in nonnormally distributed variables or those with heterogeneous variances were checked using the Mann–Whitney (two groups) or Kruskal-Wallis tests (more than two groups). For qualitative variables, the numbers (*n*) and proportions (%) were calculated and collected in cross-tables. The correlations between qualitative characteristics were assessed using Pearson's chi-squared test, with *P* < 0.05 indicating a significant correlation.

## 3. Results

The study group consisted of 85 patients with an odontogenic infection in the head and neck region, 27 women (31.8%) and 58 men (68.2%), aged 5 to 72 years (*M* = 34.8; SD = 14.8). The gender and age distribution of the patients is shown in Table 1.

**Figure 1:**
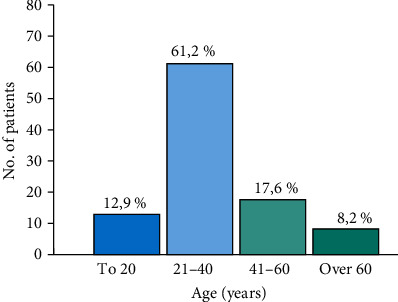
Detailed age distribution.

A detailed analysis of the patients' ages showed that 12.2% were under 20 years, 61.2% were 21-40 years, 17.6% were 41-60 years, and 8.2% were >60 years (Figure 1).

**Figure 2:**
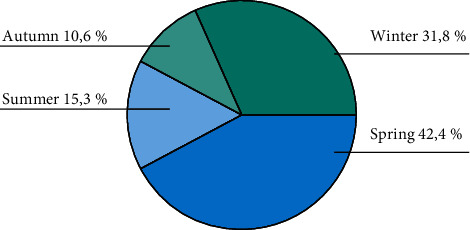
Incidence according to season.

The greatest proportion of patients were treated during the spring season (March to May; 36 patients, 42.4%), with the fewest observed in the autumn (September to November; 9 patients, 10.6%). The seasonal distribution of incidence is presented in Figure 2 and Table 2.

In terms of abscess location, the most frequently occupied areas were the submandibular (SMD) 75.3%, buccal (B) 18.8%, parapharyngeal (PPH) 15.3%, submental (SMEN) 14.1%, sublingual (SL) 11.8%, phlegmon colli (PH. COLLI) 10.6%, pterygomaxillary (PM) 4.7%, and infratemporal (ITP) 3.5% spaces. The least common abscess locations were the temporal space (TP) and the canine fossa (CF) (1.3% each).

Infection of more than one region was observed in 47.1% of the patients; infection of two sites was observed in 38.8%, of three sites in 4.7%, of four sites in 2.4%, and of five sites in 1.2%.

The most common odontogenic source of infection was the mandibular molars (74.1%), and no infections arising from the incisors and canines of the mandible were observed. A detailed distribution of infection sources according to the dental group is presented in Table 3.

All patients (100%) were treated surgically by incision and drainage of the abscess. Most procedures (71.8%) were performed under local anesthesia with pharmacological premedication. General anesthesia with endotracheal intubation was used in 28.2% of the patients.

Material for microbiological examination was also collected by incision and drainage. Aerobic bacteria were found in 36.5% of the patients, anaerobic in 16.5%, and mixed cultures in 29.4%. No pathogens were found in 17.6% of patients.

**Figure 3:**
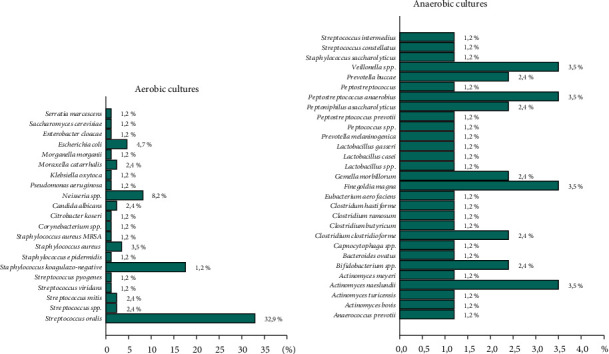
Specific distribution of the aerobic and anaerobic cultures.

Figure 3 shows a detailed distribution of the bacterial cultures. The most frequently isolated aerobic strain was *Streptococcus oralis* (32.9%), while the most common anaerobic strains were *Finegoldia magna*, *Veillonella* spp., *Peptostreptococcus anaerobius*, and *Actinomyces naeslundii* (3.5% each).

Laboratory markers of inflammation, such as leukocytosis and CRP, were evaluated. The mean leukocyte count on the first day of hospitalization was 14.4 × 10^9^/L. The mean count was higher in men (14.9 × 10^9^/L) than in women (13.3 × 10^9^/L), but the difference was not statistically insignificant. The mean CRP value was 159.8 mg/dL, being significantly higher in men (175.4 mg/dL) than in women (127.8 mg/dL).

The inflammatory markers varied with the location of infection. The highest mean lymphocyte counts were observed in patients with infections in the PM (20.3 × 10^9^/L) and M spaces (20.3 × 10^9^/L). The highest mean CRP levels were found in infections of the neck phlegmon (245.8 mg/dL). Patients with infections of the CF exhibited the lowest leukocyte (8.0 × 10^9^/L) and CRP levels (36.9 mg/dL).

A detailed distribution of laboratory markers of inflammation with the location of infection presented in Table 4.

In terms of hospitalization, most patients were hospitalized for up to 5 days (64.7%), followed by those hospitalized for 6-10 days (25.9%). Patients hospitalized for over 10 days were the smallest group.

## 4. Discussion

Despite good access to dental care, availability of antibiotic therapy, and good socioeconomic conditions, odontogenic infections of the head and neck region are still the main cause of hospitalization in maxillofacial surgery departments [1, 2, 7, 13, 31]. Infection of the deep fascial space may not manifest as a clear deterioration of general condition, which hinders early diagnosis, delays the initiation of treatment, and increases the risk of complications [7, 18, 28]. Odontogenic infections are the most common infections in the head and neck region [2, 6, 8, 32]. Prabhu et al. studied 1034 patients over 17 years and found that 78.43% of all head and neck infections were of odontogenic etiology [2]. Adoviča et al. reported that 139 of 263 (70.6%) infections in the head and neck region were odontogenic [3]. In contrast, Bakir et al. reported the lowest rates at 48.6%, noting that other common causes include peritonsillar infections (19.7%) and tuberculosis (6.9%) [8].

The head and neck form a unique anatomical region where inflammatory conditions have distinct characteristics [6, 31, 33, 34]. This is mainly related to the complex anatomy of the face and neck, the presence of teeth in the oral cavity, the vicinity of the paranasal sinuses, the rich blood supply, and the presence of vital organs responsible for sight, hearing, smell, and taste [24, 32, 33]. Inflammation is the body's defensive reaction to a damaging stimulus, which may be physical or biological (pathogenic microorganisms). In the case of a bacterial infection, the host's immune response breaks down and the infection develops. This mechanism is the main cause of inflammation in the head and neck region [9–11]. The pathogens most commonly arise from necrotic tooth pulp or tooth roots, pathologies of the apex and periodontium, infected odontogenic cysts, and pathologies associated with completely or partially retained teeth [1, 6, 14]. The infections usually start at the necrotic pulp of the molars and rarely at the premolars or single-root teeth in the anterior segment of the maxilla and mandible [3, 14, 32, 33]. Periodontitis is an etiological factor in 20-30% of odontogenic infections [34].

The second group of inflammatory conditions comprises those due to nondental causes, which include salivary gland inflammation, paranasal sinusitis, and inflammatory pathologies of the lymph nodes and skin. They can manifest clinically as inflammatory infiltrations (or cellulitis), abscesses, and more severe forms of infections such as phlegmon [19, 33, 35]. Inflammatory infiltration is an accumulation of serous exudate and elements of the plasma outside the vascular bed. The increased vessel permeability of vessels is caused by numerous locally produced mediators in response to the damaging stimulus. The inflammatory infiltration leads to board-like or pitting tissue edema, spontaneous pain and, when palpated, pain, reddening, and warming of the skin or mucous membranes. The general condition is usually moderately good, with a body temperature of up to 38°C. Depending on the bacteria's virulence, host immune status, and treatment, the inflammatory infiltration may be absorbed, spread to adjacent structures, or form an abscess as a self-limiting process [1, 4, 13, 25]. An abscess is a limited interstitial, interfascial, or soft tissue cavity filled with pus. The mature form of an abscess (abscessus maturus) is surrounded by a layer of inflammatory tissue, called the pyogenic membrane. Pus is a pathological fluid formed from plasma elements and cellular fluid with bacteria, necrotic tissue, and granulocytes. In the presence of an abscess, the general symptoms are more severe. Patients report an increased body temperature in the range of 38-39°C lasting up to several days. An increase in inflammatory markers with leukocytosis (>10 × 10^9^/L) and CRP elevation (>5 mg/dL) may be observed. Phlegmon is the most severe form of acute purulent inflammation, which involves several anatomical spaces, in contrast to abscesses, which are limited to one fascial space. Phlegmon is accompanied by very intense general symptoms, such as a fever of up to 40°C, fluid and electrolyte disturbances, and symptoms of septic shock. The condition may last from a few hours to several days. Laboratory findings show more prominent aberration than in the case of an abscess [6, 19, 31].

Ludwig's angina is a unique form of severe head and neck infection and is defined as purulent soft tissue inflammation of the floor of the mouth, with bilateral involvement of the SM, SL, SMD, and, occasionally, the PPH spaces [35, 36]. The patient's general condition is severe, accompanied by a high fever and chills. The most severe complication of Ludwig's angina is obstructive respiratory failure due to the root of the tongue exerting pressure on the posterior wall of the middle pharynx. In the first stage of the progressive dyspnea, the patient assumes a sitting position, which facilitates breathing. Patients will ultimately require urgent hospital treatment with intravenous pharmacotherapy; control of vital signs, including saturation; and securing ventilation through intubation or tracheostomy [25, 33, 35]. In addition, surgical revision and drainage of the fascial spaces involved in the inflammation, sample collection for microbiological examination, extraction of the causative teeth, and broad-spectrum empirical antibiotic therapy are required [24, 36, 37].

Odontogenic inflammations are mostly caused by a mixture of bacterial flora [12, 13, 38, 39]. Bahl et al. reported that the most frequently isolated aerobic bacteria from head and neck infections were *Streptococcus viridans* (45.0%), followed by *Staphylococcus aureus* (20.0%), coagulase-negative staphylococci (10.0%), and Corynebacterium species and *Pseudomonas aeruginosa* in 5% each. Four anaerobic bacteria were also isolated: Peptostreptococcus (20%), Bacteroides and Prevotella were found in 30% each, and Porphyromonas (5%). The researchers noted aerobic bacteria (obligate and facultative) in 25 of cases, anaerobic bacteria in 15, and aerobic-anaerobic in 60 of the cases [12]. In our study, the cultures were positive for aerobic bacteria in 36.5%, anaerobic in 16.5%, and aerobic-anaerobic in 29.4% of the cases, while in 17.6% no microorganisms were observed, which is in no agreement with Chunduri et al.'s study [20]. The most common aerobic pathogen in our study was *Streptococcus oralis* (32.9%), which belongs to the *Streptococcus* viridans group, similar to Bahl et al.'s and Chunduri et al.'s report [12, 20]. Over a longer period of infection, anaerobic flora appears due to the decreased oxidative potential and pH in inflamed tissues [38, 39].

Synthetic penicillins remain the drugs of choice in the treatment of odontogenic infections in the head and neck region [1, 9, 38]. This is mainly due to their high efficacy, minimal side effects, easy availability, and low treatment costs. Second generation *β*-lactamase cephalosporins are used almost as often as synthetic penicillins. The main second-generation cephalosporins used is cefuroxime, which exhibits a very broad spectrum of antibacterial activity and good penetration into the bone tissue, which is an asset in the treatment of odontogenic infections. The basic form of the drug is used parenterally and mainly for inpatient treatment. The drug of choice when anaerobic flora is suspected is the chemotherapeutic metronidazole [1, 12]. The indications for antibiotic therapy are severe general symptoms, extensive local inflammation, immunosuppressive states, systemic diseases (diabetes, rheumatic disease, liver damage), and inflammation of the upper and midface [1, 20]. Empirical therapy, usually using a multidrug regimen, is initiated until microbiological examination results are available (usually 72 h). The bacteriological examination is performed to identify the species of microorganism, its susceptibility to specific groups of antibiotics and chemotherapeutics (antibiogram), and to determine the MIC, which is helpful in determining the most effective drug dose [21, 38, 39].

All patients hospitalized due to an odontogenic infection were treated surgically by incision and drainage. General anesthesia with endotracheal intubation or local anesthesia with premedication was used. Respiratory obstruction and difficulties in endotracheal intubation pose great challenges in head and neck odontogenic infections. Trismus and anatomical aberrations are particularly difficult situations. Keswani et al. reported a series where all patients were treated with incision and abscess drainage under local anesthesia and analgosedation without respiratory support [39].

The location of the infection is a crucial determinant of the surgical incision site and abscess drainage. It is mainly determined by the causal teeth group and muscle attachments in a given anatomical region [2, 8, 9, 23, 24]. The most common locations of odontogenic infections according to Shah et al. were the SMD (30%), B (20%), SM (15%), and SL spaces (10%). The least frequent were the PPH (8%), CF (5%), M (4%), and PM spaces (2%) [40]. Mathew et al. investigated 137 patients with an odontogenic infection in the head and neck region and reported that the most common location was the SMD space (69.3%) [41]. Odontogenic infections may occupy several sites in the head and neck region, and the SMD space is usually the first site occupied [35, 40, 41].

In our own study, patients that were hospitalized for up to 5 days comprised the greatest proportion. This seems to indicate prompt diagnosis and effective treatment. Zheng et al. reported an average hospitalization of 8.99 ± 8.41 days in elderly group patients, decreasing to 8.73 ± 7.38 days in nonelderly group patients, with no statistical insignificance [13]. Keswani et al. reported hospitalization for 3 to 10 days in patients with an odontogenic infection in the head and neck region [39].

## 5. Conclusions

The results of our analysis show that lethal odontogenic inflammations remain a persistent problem.

Despite access to dental care and antibiotic treatment, odontogenic infections in the head and neck region are still number one in most retrospective analyses of infections in the head and neck region.

It seems that the elective elimination of odontogenic foci in dental surgeries may contribute to the reduction in complications resulting from infections at these sites as well as the need for hospitalization and hospitalization-related costs borne by the state.

It is a mistake to provide antibiotic treatment without a surgical intervention in order to remove the odontogenic cause and perform abscess drainage.

Primarily, our study aims at analysing the adult population, although odontogenic infections are problems experienced in childhood and adolescence as well.

## Figures and Tables

**Table 1:** Overall age structure.

	Valid N	Mean	Median	Minimum	Maximum	Lower quartile	Upper quartile	Standard deviation
Age	85	34.8	32	5.0	72	25	41	14.8

**Table 2:** Incidence according to season.

Season of hospitalization	No. patients (*n* = 85)	(%)
Winter	27	(31.8)
Spring	36	(42.4)
Summer	13	(15.3)
Autumn	9	(10.6)

**Table 3:** Distribution of causal dental groups.

Causal group of teeth	No. patients (*n* = 85)	(%)
Molars of the lower jaw	63	(74.1)
Molars of the upper jaw	3	(3.5)
Premolars of the lower jaw	3	(3.5)
Premolars of the upper jaw	1	(1.2)
Incisors and canines of the lower jaw	0	(0.0)
Incisors and canines of the upper jaw	1	(1.2)
Many causal groups of teeth	13	(15.3)
No data	1	(1.2)

**(a):** 

	Total	Women	Men	*P* value
Leukocytes (×10^9^/L):	*n* = 84	*n* = 27	*n* = 57	0.478
Mean ± SD	14.4 ± 5.2	13.3 ± 3.5	14.9 ± 5.8	
Median [Q1; Q3]	13.5 [10.9; 16.7]	13.6 [10.7; 15.5]	13.4 [11.0; 18.7]	
Min-max	5.7–30.2	6.6–21.0	5.7–30.2	
CRP (mg/dL):	*n* = 82	*n* = 27	*n* = 55	**0.041**
Mean ± SD	159.8 ± 109.6	127.8 ± 101.6	175.4 ± 110.9	
Median [Q1; Q3]	142 [71; 216]	126 [60; 169]	176 [86; 235]	
Min-max	15–521	15–521	20–451	

**(b):** 

Location	Leukocytes (×10^9^/L)	CRP (mg/dL)
SMD	*n* = 63	*n* = 61
Mean ± SD	14.7 ± 5.4	169.1 ± 116.7
Median [Q1; Q3]	14 [11; 18]	158 [71; 234]
Min-max	6–30	20–521
SMEN	*n* = 12	*n* = 12
Mean ± SD	11.8 ± 3.0	136.3 ± 103.9
Median [Q1; Q3]	12 [10; 14]	105 [66; 185]
Min-max	6–16	34–375
SL	*n* = 9	*n* = 9
Mean ± SD	13.2 ± 4.3	132.1 ± 126.6
Median [Q1; Q3]	11 [10; 14]	80 [63; 119]
Min-max	9–23	34–375
ITP	*n* = 3	*n* = 3
Mean ± SD	14.1 ± 7.1	186.7 ± 71.9
Median [Q1; Q3]	12 [8; 22]	154 [137; 269]
Min-max	8–22	137–269
TP	*n* = 1	*n* = 1
Mean ± SD	8.5 ± 0.0	269.1 ± 0.0
Median [Q1; Q3]	8 [8; 8]	269 [269; 269]
Min-max	8–8	269–269
PPH	*n* = 13	*n* = 13
Mean ± SD	16.3 ± 5.3	128.8 ± 63.7
Median [Q1; Q3]	15 [12; 19]	146 [80; 161]
Min-max	9–26	36–234
B	*n* = 16	*n* = 16
Mean ± SD	16.0 ± 5.6	161.3 ± 121.3
Median [Q1; Q3]	15 [13; 19]	125 [76; 215]
Min-max	7–29	15–451
PH. COLLI	*n* = 8	*n* = 8
Mean ± SD	16.6 ± 8.8	245.8 ± 126.9
Median [Q1; Q3]	14 [10; 25]	251 [175; 321]
Min-max	6–30	26–445
PM	*n* = 3	*n* = 3
Mean ± SD	20.3 ± 7.6	231.5 ± 93.7
Median [Q1; Q3]	23 [12; 26]	234 [137; 324]
Min-max	12–26	137–324
CF	*n* = 1	*n* = 1
Mean ± SD	8.0 ± 0.0	36.9 ± 0.0
Median [Q1; Q3]	8 [8; 8]	37 [37; 37]
Min-max	8–8	37–37
M	*n* = 3	*n* = 3
Mean ± SD	20.3 ± 8.3	214.1 ± 20.6
Median [Q1; Q3]	24 [11; 26]	216 [193; 234]
Min-max	11–26	193–234

Q1: lower quartiles; Q3: upper quartiles; SD: standard deviation; CRP: c-reactive protein; SMD: submandibular; B: buccal; PPH: parapharyngeal; SMEN: submental; SL: sublingual; PH. COLLI: phlegmon colli; PM: pterygomaxillary; ITP: infratemporal; TP: temporal; CF: canine fossa; M: masseter.

## Data Availability

The clinical data used to support the findings of this study may be released upon application to the Department of Maxillofacial Surgery, Silesian Piast Medical University, Borowska 213, Wrocław 50-556, Poland, who can be contacted through Ewa Zawiślak (Ewazawislak0@op.pl).
